# Comparative Surface Electrostatics and Normal Mode Analysis of High and Low Pathogenic H7N7 Avian Influenza Viruses

**DOI:** 10.3390/v15020305

**Published:** 2023-01-21

**Authors:** Giulia Baggio, Francesco Filippini, Irene Righetto

**Affiliations:** Synthetic Biology and Biotechnology Unit, Department of Biology, University of Padua, Via Ugo Bassi, 58/B, 35131 Padua, Italy

**Keywords:** influenza A virus, LPAI, HPAI, electrostatic distance, normal modes analysis, fingerprint, pandemic virus, SARS-CoV-2

## Abstract

Influenza A viruses are rarely symptomatic in wild birds, while representing a higher threat to poultry and mammals, where they can cause a variety of symptoms, including death. H5 and H7 subtypes of influenza viruses are of particular interest because of their pathogenic potential and reported capacity to spread from poultry to mammals, including humans. The identification of molecular fingerprints for pathogenicity can help surveillance and early warning systems, which are crucial to prevention and protection from such potentially pandemic agents. In the past decade, comparative analysis of the surface features of hemagglutinin, the main protein antigen in influenza viruses, identified electrostatic fingerprints in the evolution and spreading of H5 and H9 subtypes. Electrostatic variation among viruses from avian or mammalian hosts was also associated with host jump. Recent findings of fingerprints associated with low and highly pathogenic H5N1 viruses, obtained by means of comparative electrostatics and normal modes analysis, prompted us to check whether such fingerprints can also be found in the H7 subtype. Indeed, evidence presented in this work showed that also in H7N7, hemagglutinin proteins from low and highly pathogenic strains present differences in surface electrostatics, while no meaningful variation was found in normal modes.

## 1. Introduction

Influenza A viruses can infect both birds and mammals, representing an economic threat to poultry farming, where millions of birds are routinely culled to block transmission and, less frequently, dangerous risk to human health. The worst (historically recorded) pandemic event in humans was the H1N1 influenza of 1918 (Spanish Flu: 20–100 million deaths worldwide, depending on estimates), while the H2N2 (1957) and the H3N2 (1968) pandemics caused 1 million deaths, i.e., two/threefold the baseline yearly death toll associated with seasonal influenza [1,2,3,4]. “Highly pathogenic avian influenza” (HPAI) and “low pathogenic avian influenza” (LPAI) were first defined to classify flu viruses in 1981, in the 1^st^ International Symposium on Avian Influenza [5]. In 1997, a poultry to human host jump occurred with H5N1 in Hong Kong [6].

HPAI are found only among H5 and H7 subtypes, and these strains arise from LPAI ones; no naturally transmitted virus of the other subtypes has a mortality rate high enough to meet the classification criteria of highly pathogenic [1]. Zoonotic infections of humans by H5 and H7 viruses can result in pneumonia, acute respiratory distress syndrome (ARDS) and even death [7,8]. However, if an H5/H7 virus were to evolve the ability for direct, human-to-human airborne transmission, this could lead to devastating pandemic events like the Spanish flu [9], and this makes it extremely important to identify molecular fingerprints underlying host jump and pathogenicity shift.

The two major capsid proteins, hemagglutinin (HA) and neuraminidase, play a major role in the interaction of avian influenza (AI) viruses and host cells, as HA binds sialic acid (SA) as a functional receptor, and HA linkage to either α2,3-linked SA (avian) or α2,6-linked SA (mammalian/human) is a well-known host determinant [10,11,12].

However, sequence-based prediction of SA-binding efficacy is difficult, because even a few mutations may change HA specificity. Neuraminidase can also influence host specificity and respiratory-droplet transmissibility [13]. HA is also crucial to AI pathogenicity; however, other surface proteins such as neuraminidase and M1 also play a role, and even variation of internal proteins such as the NP can influence the pathogenicity [14]. Indeed, the pathogenic potential of a virus is a complex matter, as demonstrated by a study on rebuilding of the 1918 Spanish flu: full virulence potential could be achieved only with all of the original eight gene segments together, and any replacement lowered the virulence in mice [15].

A molecular signature for the LPAI to HPAI shift is an increased number of basic amino acids in the HA polybasic cleavage site or polybasic motif [16]. Changes in the surface features of HA have been reported to play an important role in the evolution and spreading of both H5 and H9 subtype clades [17,18], as well as in modulation of AI host specificity [19,20,21,22], while their influence on pathogenic shift is still unclear.

The recent finding in H5N1 of surface fingerprints specific to LPAI or HPAI [23] prompted us to extend the investigation and check whether such pathogenicity-related fingerprints are restricted to the H5 subtype, or can also be found in H7, possibly representing a pathogenicity hallmark of AI viruses. Comparative analysis of surface electrostatics has been able to identify fingerprints in clade evolution [17,18], as well as to suggest profiles in host specificity [24]. When surface electrostatics was also applied for the comparative analysis of LPAI and HPAI in H5N1, once again it was able to suggest fingerprints; moreover, normal mode analysis (NMA) provided further information, as fluctuation profiles unveiled an intriguing LPAI/HPAI difference at the 110-helix region [23].

Results from this work seem to confirm in H7 subtype that LPAI and HPAI show differences in the electrostatic profiles, while no meaningful variation was unveiled by normal modes analysis.

## 2. Materials and Methods

### 2.1. Structure Modeling, Refinement, Assessment, and Comparison

HA protein sequences were obtained from the NIAID Influenza Research Database (IRD) [25], which is available online [26], and subsequently screened for keeping only non-redundant sequences.

The target protein sequences were modeled by homology modeling using MODELLER [27,28] on the best available structure templates. Two models were built for each sequence; then, the GA341 score [29], which uses percentage of sequence identity between template and model as parameter, and the discrete optimized protein energy (DOPE) score [30], which assesses the energy function, were calculated for each model in order to assess its quality. Due to the very high identity between all templates and models, the lowest DOPE score was selected as a discriminant in choosing the best model.

Refinement of the structures was performed through SCWRL4 [31], which integrates a rotamer library, a frequency-dependent energy function based on rotamer frequencies and a repulsive steric energy term, and a graph decomposition in order to calculate the optimal side-chain packing. Finally, each model quality was checked via the QMEAN server [32] using the QMEAN6 composite scoring function [33]. Refined protein structures were viewed using the UCSF Chimera 1.16 software [34].

PDBeFold [35] was used to perform structural alignment through rigid superposition and to calculate root mean square deviation (RMSD), identity, and Q-score between structures.

### 2.2. Electrostatic Potential Analysis

Comparative analysis of electrostatic potentials was performed with the Adaptive Poisson–Boltzmann Solver (APBS) [36] at the APBS-PDB2PQR software suite web server [37]. PDB2PQR automates preparation of structures for subsequent analysis, adding atomic charge and radius parameters to PDB data and estimating titration states and protonation. APBS solves the equations of continuum electrostatics for biomolecular assemblages, calculating electrostatic and solvation properties, through the implicit solvent model Poisson–Boltzmann (PB), which provides a global solution suited to visualization and structural analyses. Parameters were chosen according to the previous literature [17,18,23]. 

Spatial distribution of the electrostatic potential was calculated at physiological ionic strength (I) = 150 mM, assuming +1/−1 charges for the counter-ions. Partial charges and van der Waals radii were assigned via PDB2PQR according to the PARSE force field [38], choosing an interior $\varepsilon_{p}$ = 2 for proteins and $\varepsilon_{s}$ = 78 for solvent. Temperature was set at T = 298.15 K. Probe radius for dielectric surface and ion accessibility surface were set to be r = 1.4 Å and r = 2.0 Å. Isopotential contours were plotted at ±1 kBT/e and viewed using UCSF Chimera [34]. 

Electrostatic distance (ED) matrices were calculated by protein interaction property similarity analysis (PIPSA) [39] with Hodgkin indexes at the webPIPSA server [40]. PIPSA provides calculation of the protein electrostatic potentials, calculates similarity indices for all pairs of proteins based on the electrostatic similarity, and converts the similarity indices into electrostatic “distances” that are visualized through heat maps and epograms (tree-like diagrams based on electrostatic differences). The ED between two molecules a and b is calculated with the following equation:

$$ED_{a,b}=\sqrt{2-2SI_{a,b}}\;\;$$

where the Hodgkin similarity index (SI) is provided in turn by:

$$SI_{a,b}=\frac{2\left(p_{a},p_{b}\right)}{\left(p_{a},p_{a}\right)+\left(p_{b},p_{b}\right)}$$

where (*p_a_*, *p_b_*), (*p_a_*, *p_a_*), (*p_b_*, *p_b_*) are the scalar products of the electrostatic potentials over the region where the potentials are compared. Visualization of the clusters and generation of the trees were either provided by webPIPSA or performed by the statistical program R [41], which is based on webPIPSA calculations.

### 2.3. Normal Modes Analysis (NMA)

Protein structures used as templates for modeling were retrieved from the Protein Data Bank (PDB) at the RCSB PDB website [42]. Structures used for modeling target H7N7 HA sequences were PDB structures 4DJ6 (resolution 2.61 Å), from strain A/Netherlands/219/2003 (H7N7), and, at the beginning of this work, 6N5A (resolution 3.30 Å), from strain A/equine/NY/49/73 (H7N7), which was then replaced by 7T1V as an HA structure from the same strain, but with improved resolution (2.05 Å).

High confidence homology modeling was performed as illustrated above; however, five models were created for each protein sequence, and a very thorough optimization with the variable target function method (VTFM) and slow molecular dynamics (MD) and simulated annealing (SA) were performed, completing a total of 300 iterations of VTFM optimization. Model refining with SCWRL4 and quality checking through QMEAN were performed as previous methodological set-ups.

Single and comparative NMA analyses were performed with WebNM@ [43], which uses the coarse-grained elastic network model (ENM) of a pdb file to calculate low-frequency normal modes of single pre-aligned protein structures or sets thereof. ENM is a reliable and computationally cost-effective way to analyze protein flexibility and dynamics as well as to highlight differences between protein structures that normal comparison, such as the RMSD, may overlook.

The protein is represented as a string of $C_{\alpha }$ atoms, and the interaction between two atoms is described by the pair potential $U_{ij}$, as shown in the following equation:

$$U_{ij}\left(R\right)=k\left(\left|R_{ij}{}^{0}\right|\right)(\left|R_{ij}\right|-{\left|R_{ij}{}^{0}\right|}^{2})$$

where *ri* and *rj* are the positions of residues *i* and *j* in the current conformation of the protein, the superscript 0 denotes the equilibrium conformation, and *k* is the force constant for the spring connecting residues *i* and *j*.

In single mode, dynamic cross-correlation matrices (DCCMs) are calculated, which help identify correlated and anticorrelated protein motions. DCCMs are two-dimensional matrices with a value range from 1 for completely correlated motions to −1 for anticorrelated motion. Coupling between two $C_{\alpha }$ atoms *i* and *j* in the DCCM is given by the following equation:

$$C_{ij}=\frac{\sum_{m=1}^{M}\left[X_{m}\right]i\left[X_{m}\right]j}{\sqrt{\sum_{m=1}^{M}\frac{1}{y_{m}}\left[X_{m}\right]i\left[X_{m}\right]i\;}\sqrt{\sum_{m=1}^{M}\frac{1}{y_{m}}\left[X_{m}\right]j\left[X_{m}\right]j\;}}$$

where $X_{m}$ and $y_{m}$ represent the eigenvectors and eigenvalues of the $m^{th}$ normal mode. Default settings for WebNM@ were used according to previous work [23].

Dynamic similarity was also investigated through the Bhattacharyya coefficient (BC) and root mean square inner product (RMSIP). BC values range from 0 to 1, with 1 being the maximum overlap between the collective dynamics of the aligned proteins. RMSIP is a measure of the similarity between two sets of modes, calculated from the $C_{\alpha }$ atom fluctuations for the lowest normal modes, as follows:

$$RMSIP=\sqrt{\frac{1}{n}\left[\overset{n}{\underset{i=1}{\sum }}\overset{n}{\underset{j=1}{\sum }}{\left(X_{i}Y_{i}\right)}^{2}\right]}$$

RMSIP ranges from 0 to 1, with 1 being the maximum similarity in $C_{\alpha }$ fluctuations. WebNM@ further provides a graph of normalized $C_{\alpha }$ fluctuations calculated as the sum of the $C_{\alpha }$ atom displacements in each mode, weighted by the inverse of their corresponding eigenvalues. WebNM@ utilizes the first 200 modes to calculate the graph, from which flexible protein regions can be inferred by inspecting the peaks of the graph.

## 3. Results

### 3.1. Preliminary Comparison of a Mixed Dataset of H7N7 Hemagglutinin Proteins 

A total of 247 H7N7 HA sequences were collected from UniProtKB [44], the Influenza Research database [26], and GISAID [45]. LPAI sequences were massively overrepresented with a total of 233 sequences, while only 14 HPAI sequences could be found, of which only 12 were from different outbreaks. Redundant sequences and sequences from the same outbreak were not utilized in the same set.

Structural models were obtained via homology modeling with high confidence, due to the high identity value between template and targets, with an average identity of 95.0% between targets and 4DJ6 and of 83.9% between targets and 6N5A/7T1V. Models were further refined, finally showing no atom clashes. QMEAN analysis showed extremely high-quality values for all refined models, with the GLOBAL quality score ranging between 0.86 and 0.91, with an uncertainty of 0.06.

A preliminary analysis of root mean square deviation (RMSD) was performed with PDBeFOLD, which provides structural alignments, calculates RMSD, and identities percentages. Two sub-datasets were used, namely low and highly pathogenic strains, for comparison. 

Sequence identities, RMSD and Q-scores calculated for the HA receptor binding domain (RBD) from HPAI and LPAI H7N7 viruses are reported in Table 1 and Table 2.

RMSD and identity analysis were performed to show that, in accordance with previous research, eventual fingerprints specific to low or high pathogenicity are based on very minor sequence/structure changes and surface features, rather than on relevant structural differences.

**Figure 1:**
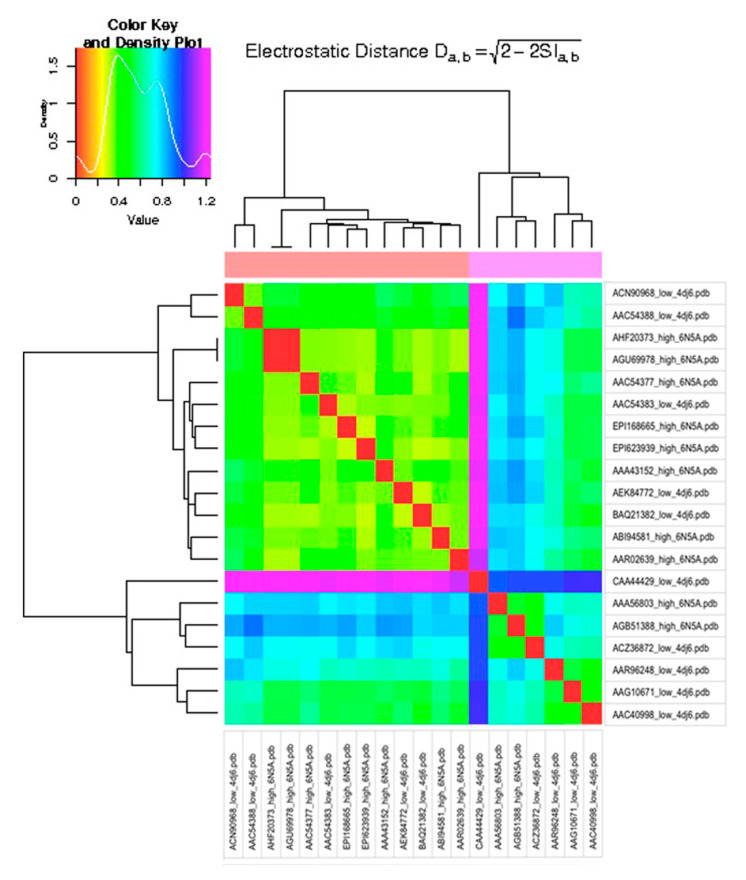
Electrostatic distance heatmap for single template (PDB 4DJ6) models.

**Figure 2:**
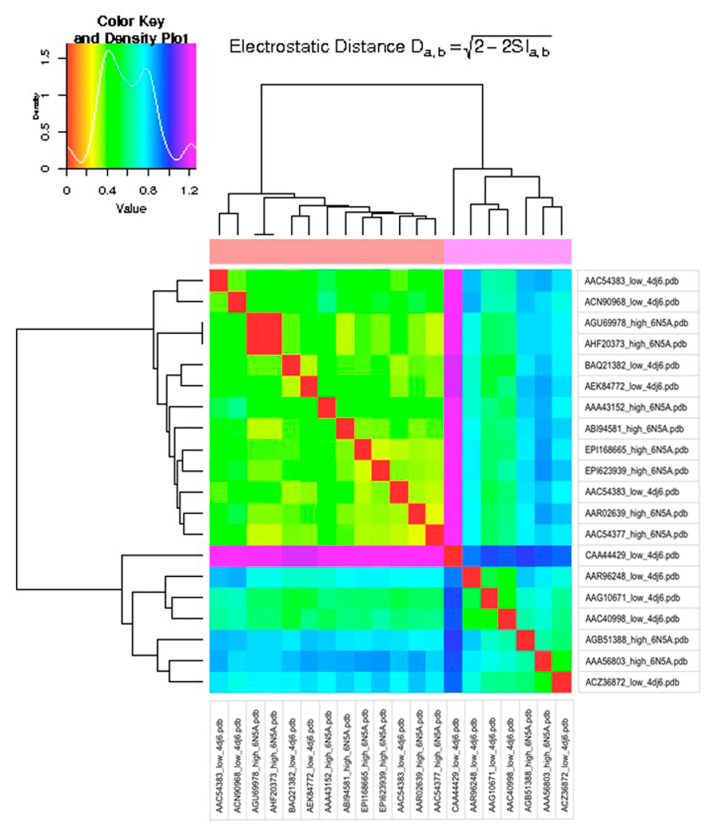
Electrostatic distance heatmap for mixed template models.

An RMSD value under 1 Å is commonly considered a cut-off for different structures [46], and RMSD values in Figure 1 and Figure 2 are largely under 1 Å and very close to 0, indicating a very strong structural conservation. This is not surprising, while being in agreement with a similarly very high sequence identity. Since the RBD is the most variable part of HA, even higher RMSD values were obtained when comparing the whole HA, whose stalk sequence is largely identical and less subjected to mutations. Overall RMSD was 0.1365 for structure 4DJ6, with an overall Q-score of 0.9979; overall RMSD for structure 6N59 was 0.1314, with an overall Q-score of 0.9981.

### 3.2. Surface Electrostatics of HA RBDs from HPAI and LPAI Strains of H7N7

According to previous work [17,18,23,24], a comparative electrostatic analysis of 20 representative RBD structures (from 10 LPAI and 10 HPAI H7N7 viruses) was performed. The spatial distribution of the electrostatic potential was calculated at I = 150 mM, and +1/−1 charges were assumed for counter-ions. Partial charges and van der Waals radii were assigned with PDB2PQR, then the APBS webserver was used to carry out calculations with the Poisson–Boltzman (PB) method. Electrostatic distance (ED) was calculated at the WebPIPSA (Protein Interaction Property Similarity Analysis) server, obtaining, for each protein, a matrix of pairwise similarity indices visualized through a heatmap.

Calculations were performed on mixed model structures (6N5A for HPAI and 4DJ6 for LPAI strains) and on single model structures (each structure modeled with 6N5A and 4DJ6) using both Hodgkin and Carbo indexes. The heatmap for structures modeled with single template 4DJ6 is presented in Figure 1 (15), while the heatmap for mixed models is presented in Figure 2 (16). 

Both heatmaps in Figure 1 and Figure 2 were built using the Hodgkin index, as no difference was found when using the Carbo index, and hence, the corresponding heatmaps are not shown.

In a previous study, intriguing differences between HA RBDs from high and low pathogenicity H5N1 strains were found, both in terms of total net charge and isocontour [23]. The difference in net charge among HPAI and LPAI H5N1 viruses was not just dependent on the positively charged residues at the HA polybasic cleavage site of HPAI, as this region is not part of the RBD, and hence, was excluded from the models. The vestigial esterase subdomain (VED) and the 110-helix region were found to show the most evident difference between the two subgroups, and this difference in the 110-helix was confirmed at the fluctuation profile level, using normal mode analysis [23].

**Figure 3:**
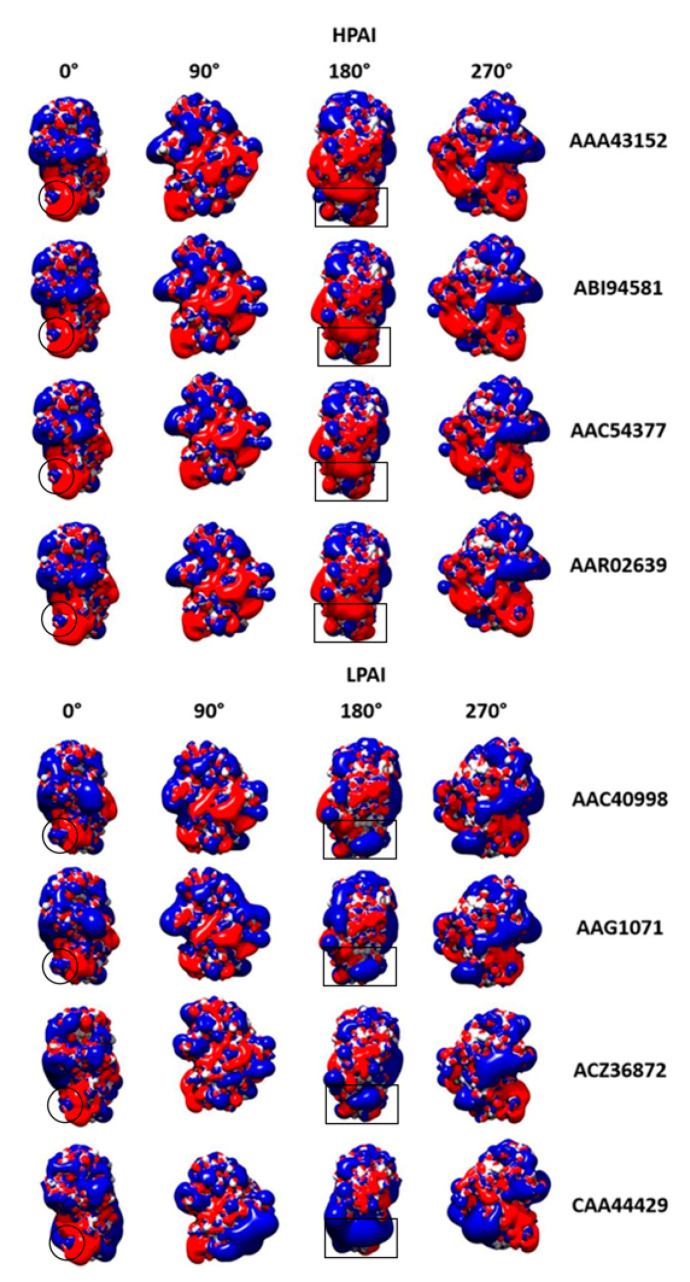
Surface charge distribution of the HA RBDs from four representative HPAI and four LPAI H7N7 strains. Positive, blue; negative, red; neutral, white. Four 90° stepwise orientations are shown. Open circle, VED subregion; open rectangle, region where K122E, K182N mutations result in negativization/depositivization.

Figure 3 highlights differences in the electrostatic isocontours of eight representative H7N7 strains: four HPAI and four LPAI. Four 90° stepwise orientations are shown, as differences may occur on each different “side” of the RBD.

The VED region, framed by an open circle (see 0° view) for comparison to H5 subtype, does not show in H7N7 an LPAI-HPAI difference as evident as that found in H5N1. In the framed VED subregion, both H7 LPAI and HPAI have a mix of negative and positive charges, like the H5N1 LPAI. 

However, two LPAI to HPAI mutations, K122E and K182N, result in the evident negativization/ depositivization of a large RBD surface (framed in the figure by an open rectangle, see 180° view), clearly becoming more negative (red) in HPAI. Such a fingerprint is differentially conserved in H7N7 viruses, both in the eight representative HPAI and LPAI depicted in Figure 3, and in the other HPAI and LPAI H7 viruses that are not shown. 

Intriguingly, mutation K122E, where charge shifts from +1 to −1, occurs at the most C-terminal residue of the 110-helix, with further depositivization being favored by close mutation K182N.

### 3.3. Normal Mode Analysis

NMA of HA RBDs from LPAI and HPAI H7N7 strains was performed using WebNM@. To avoid statistical bias, 10 different sets of 20 RBD sequences were used (the maximum amount that can be compared with WebNM@ due to computational constraints), each with 10 LPAI and 10 HPAI (randomly selected).

Normal modes were analyzed by the Bhattacharyya coefficient (BC) heatmap, root mean square inner product (RMSIP), dynamic cross-correlation matrices (DCCM) and finally with fluctuation analysis of single amino acids.

**Figure 4:**
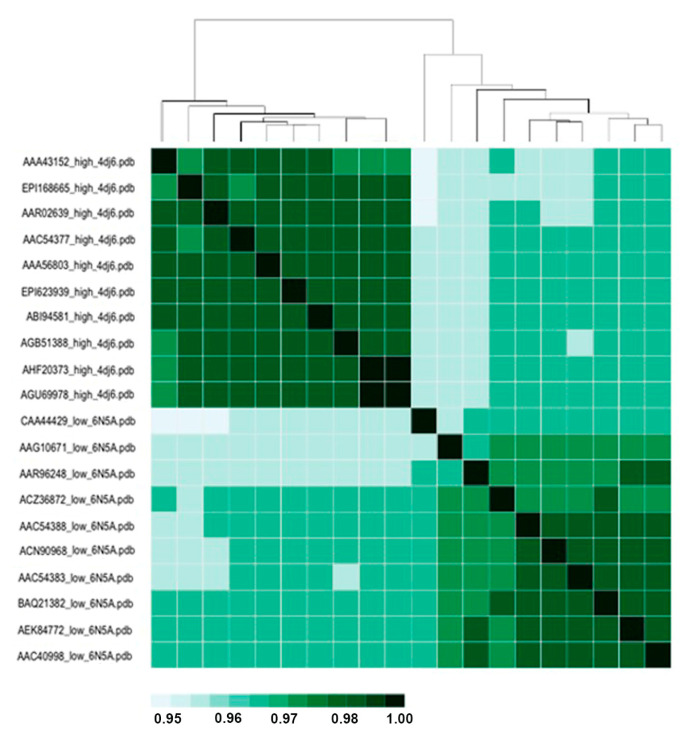
Bhattacharyya coefficient (BC) heatmap for HPAI and LPAI RBDs. Uniprot AC for each sequence, followed by pathogenicity and PDB of the template, are reported on the Y axis, while a distance tree is reported on the X axis.

**Figure 5:**
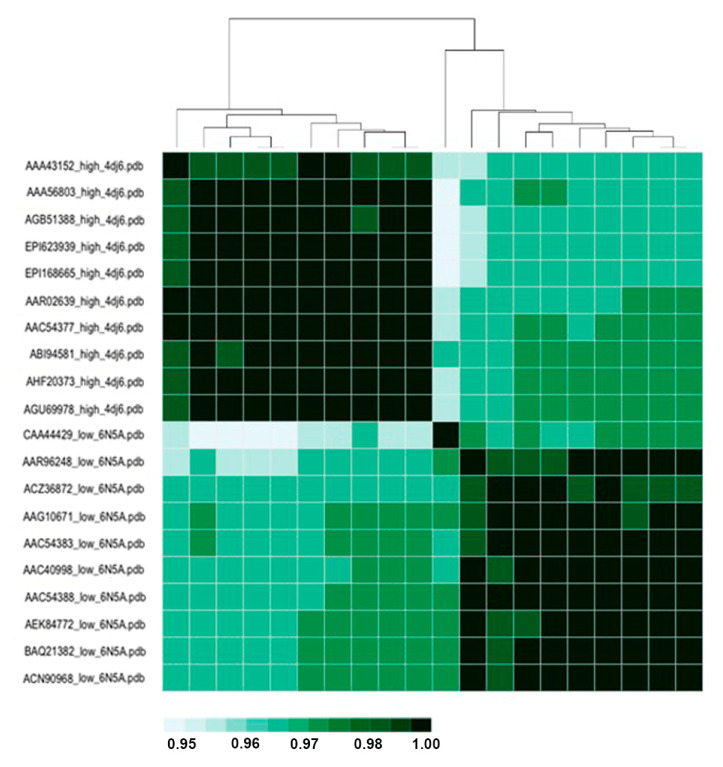
RMSIP heatmap for HPAI and LPAI RBDs. Uniprot AC for each sequence, followed by pathogenicity and PDB of the template are reported on the Y axis, while a distance tree is reported on the X axis.

As shown by the BC and RMSIP of the 20 chosen sequences, presented in Figure 4 and Figure 5, respectively, the structures sharply segregate across pathogenicity (rather than based on phylogenetic distance, not shown). The color code varies from lighter green (higher divergency) to darker green (closest structures).

However, such results might only apparently be positive. 

Indeed, both the BC and RMSIP present a high grade of closeness for all structures, ranging from 0.95 to 1, so we can infer that the dynamics between LPAI and HPAI viruses vary marginally in H7N7, even less than in H5N1, where the reported coefficient is 0.92 [23]. Moreover, models for LPAI and HPAI were obtained using two different templates, and hence, sorting might depend on differences in the template rather than on pathogenicity-related variation. 

Therefore, to check the validity of the NMA clustering, and according to previous work, all 20 sequences were modeled on a single template, either 4DJ6 or 6N5A, and this caused pathogenicity-specific sorting to be lost (not shown), suggesting NMA is not applicable to this H7 subtype dataset, possibly because of the aforementioned high closeness values. 

Nevertheless, correlation matrices were also calculated for each sequence, and representative pairs for LPAI and HPAI were chosen for visualization purposes. In all instances, correlation matrices were found to be nearly identical, with strongly correlated motions, occurring through the RBD, and weak anticorrelated motions (not shown).

When focusing on the 110-helix, as both an important determinant in the VED region of the hemagglutinin and the region having shown LPAI-HPAI variation in H5N1 [23], once again, no meaningful difference could be highlighted (not shown).

## 4. Discussion

Sequence divergence does not always correspond to structural divergence; two proteins with high sequence similarity may show very different properties considering recognition of epitopes by specific antibodies, and two proteins may share antigenic properties even with highly divergent sequences if epitopes are conserved. Local changes in accessible surface area, electrostatic potential, hydropathy or hydrophilicity features can change motif functionality.

Sequence-based studies are fundamental to highlight conservation—and differences—of amino acids close in sequence, but are limited in highlighting “group variation”, namely, identifying relationships of multiple mutations, especially when they are far from each other in primary sequence but close in 3D protein structure.

Surface features of biomolecules such as proteins are crucial to finely tune their interactions, and previous work demonstrated that comparative electrostatic and normal mode analyses can unveil functional fingerprints.

Such analyses, performed in this work, used the RBD for comparison as it is the most variable HA region, hence the best candidate to highlight differences across strains. The proteolytic and glycosylation sites were deliberately excluded, as differences at these sites between LPAI and HPAI are already known and reported. Difference in the cleavage site is extensively reported in the literature as the main discriminant for predicting pathogenicity other than direct infection of birds; however, despite its importance, it is unable to predict pathogenicity alone.

RBD “charge redistribution” has been found as a general feature of AI virus evolution and spread. This hallmark was first observed in a 2014 study [17] concerning H5N1 clades, with a denegativization of the VED associated by negativization (or depositivation) of the RBD in still-circulating clades. Due to the strong antigenic feature of both the RBD and VED subdomains, and the fact that local charge concentration is typical for antigenic epitopes, it was speculated that charge redistribution might have contributed to antigenic escape, and hence to their evolutionary success and spread.

The study was replicated in H9N2 viruses [18], with in-depth analysis that revealed structural agreement between H5 and H9 observations, showing similarity between the two HA subtypes.

Charge redistribution and mutation especially occurred in sub-regions important to sialic acid binding, host specificity and immune escape/antigenic drift: 110-helix, 130-loop, 190-helix and 220-loop. Intriguingly, variation at the 110-helix was then found to be also a potential fingerprint for LPAI-HPAI difference in H5N1, and this prompted us to check whether this would also happen with H7N7. The rationale for this is that H5, H9 and H7 viruses are studied as potential pandemic agents, all having been reported to infect mammals and humans; H5 and H7 in particular, can develop HPAI strains.

However, two main problems complicated this analysis when performed with H7. First, a general and structured clade classification for H7N7 is still missing; second, <250 non-redundant, complete nucleotide sequences of H7N7 HA are available at the Influenza Research Database, compared to >6500 H5N1 and >1600 H9N2 sequences. This notwithstanding, results from this work seem to confirm, also in the H7 subtype, that comparative electrostatic analysis can highlight meaningful differences and suggest fingerprints.

Recently, the infectivity and immune escape of another pandemic virus, SARS-CoV-2, were also found to be influenced by variation in surface electrostatics of the spike protein, as reported by comparative analysis of the SARS-CoV-2 variants [47,48,49].Lessons from comparative studies on hemagglutinin and spike protein features suggest that variation in surface electrostatics of the capsid proteins that mediate binding to the host cell functional receptors is a driving force and a functional fingerprint in the evolution of pandemic viruses.

Concerning NMA, negative results with H7 might depend on the poor dataset of sequences, on their very high structural closeness, as well as on bias derived from a very limited number of template structures for modeling. To clarify these aspects, further research entailing the use of a more complete dataset is needed.

## 5. Conclusions

Surveillance of influenza A virus is crucial, in terms of human and animal health and economy. This work sought to shed light on H7N7 influenza virus hemagglutinin fingerprints. Using an established computational framework, we were able to find some fingerprints. In particular, we noticed some mutations in the shift from LPAI to HPAI, able to change the shape of isocontour in the visual inspection. As for the H5N1 virus, electrostatic clustering sharply splits H7N7 in LPAI and HPAI strains. Moreover, H7N7 exhibits a behavior similar to that of H5N1: the RBD is subjected to “negativization” and “depositivization” events. Again, we can infer that charge redistribution may be involved in pathogenicity and immune escape

This work suggests that the residue 122 near the antigenic site 110-helix and the residue 182 may be involved in the pathogenicity shift from LPAI to HPAI. These findings can be useful for surveillance activity and vaccine design.

## Figures and Tables

**Table 1:** Sequence identity, RMSD and Q-score for LPAI sequences modeled on PDB 4DJ6.

				Sequence Identity							
	structure:	1	2	3	4	5	6	7	8	9	10
**1**	AAC40998_low_4dj6.pdb:		0.976	0.961	0.966	0.863	0.951	0.922	0.966	0.966	0.859
**2**	AAC54383_low_4dj6.pdb:	0.976		0.971	0.966	0.859	0.961	0.922	0.966	0.966	0.849
**3**	AAC54388_low_4dj6.pdb:	0.961	0.971		0.976	0.854	0.976	0.907	0.966	0.966	0.849
**4**	AAG10671_low_4dj6.pdb:	0.966	0.966	0.976		0.849	0.966	0.907	0.961	0.961	0.844
**5**	AAR96248_low_4dj6.pdb:	0.863	0.859	0.854	0.849		0.854	0.854	0.854	0.859	0.815
**6**	ACN90968_low_4dj6.pdb:	0.951	0.961	0.976	0.966	0.854		0.907	0.956	0.956	0.834
**7**	ACZ36872_low_4dj6.pdb:	0.922	0.922	0.907	0.907	0.854	0.907		0.907	0.907	0.844
**8**	AEK84772_low_4dj6.pdb:	0.966	0.966	0.966	0.961	0.854	0.956	0.907		0.990	0.849
**9**	BAQ21382_low_4dj6.pdb:	0.966	0.966	0.966	0.961	0.859	0.956	0.907	0.990		0.849
**10**	CAA44429_low_4dj6.pdb:	0.859	0.849	0.849	0.844	0.815	0.834	0.844	0.849	0.849	
				**RMSD**							
	**structure:**	**1**	**2**	**3**	**4**	**5**	**6**	**7**	**8**	**9**	**10**
**1**	AAC40998_low_4dj6.pdb:		0.123	0.100	0.129	0.109	0.113	0.125	0.118	0.118	0.156
**2**	AAC54383_low_4dj6.pdb:	0.123		0.143	0.135	0.150	0.149	0.160	0.153	0.150	0.176
**3**	AAC54388_low_4dj6.pdb:	0.100	0.143		0.107	0.104	0.106	0.114	0.126	0.132	0.151
**4**	AAG10671_low_4dj6.pdb:	0.129	0.135	0.107		0.116	0.106	0.124	0.132	0.136	0.173
**5**	AAR96248_low_4dj6.pdb:	0.109	0.150	0.104	0.116		0.098	0.114	0.128	0.146	0.177
**6**	ACN90968_low_4dj6.pdb:	0.113	0.149	0.106	0.106	0.098		0.093	0.123	0.140	0.166
**7**	ACZ36872_low_4dj6.pdb:	0.125	0.160	0.114	0.124	0.114	0.093		0.128	0.150	0.168
**8**	AEK84772_low_4dj6.pdb:	0.118	0.153	0.126	0.132	0.128	0.123	0.128		0.095	0.195
**9**	BAQ21382_low_4dj6.pdb:	0.118	0.150	0.132	0.136	0.146	0.140	0.150	0.095		0.183
**10**	CAA44429_low_4dj6.pdb:	0.156	0.176	0.151	0.173	0.177	0.166	0.168	0.195	0.183	
				**Q-score**							
	**structure:**	**1**	**2**	**3**	**4**	**5**	**6**	**7**	**8**	**9**	**10**
**1**	AAC40998_low_4dj6.pdb:		0.998	0.999	0.998	0.999	0.999	0.998	0.998	0.998	0.997
**2**	AAC54383_low_4dj6.pdb:	0.998		0.998	0.998	0.997	0.998	0.997	0.997	0.998	0.997
**3**	AAC54388_low_4dj6.pdb:	0.999	0.998		0.999	0.999	0.999	0.999	0.998	0.998	0.997
**4**	AAG10671_low_4dj6.pdb:	0.998	0.998	0.999		0.999	0.999	0.998	0.998	0.998	0.997
**5**	AAR96248_low_4dj6.pdb:	0.999	0.997	0.999	0.999		0.999	0.999	0.998	0.998	0.997
**6**	ACN90968_low_4dj6.pdb:	0.999	0.998	0.999	0.999	0.999		0.999	0.998	0.998	0.997
**7**	ACZ36872_low_4dj6.pdb:	0.998	0.997	0.999	0.998	0.999	0.999		0.998	0.998	0.997
**8**	AEK84772_low_4dj6.pdb:	0.998	0.997	0.998	0.998	0.998	0.998	0.998		0.999	0.996
**9**	BAQ21382_low_4dj6.pdb:	0.998	0.998	0.998	0.998	0.998	0.998	0.998	0.999		0.996
**10**	CAA44429_low_4dj6.pdb:	0.997	0.997	0.997	0.997	0.997	0.997	0.997	0.996	0.996	

**Table 2:** Sequence identity, RMSD and Q-score for HPAI sequences modelled on PDB 6N5A.

			Sequence Identity								
	structure:	1	2	3	4	5	6	7	8	9	10
**1**	AAA43152_high_6N5A.pdb:		0.878	0.907	0.917	0.917	0.883	0.902	0.902	0.912	0.912
**2**	AAA56803_high_6N5A.pdb:	0.878		0.898	0.912	0.917	0.976	0.898	0.898	0.898	0.898
**3**	AAC54377_high_6N5A.pdb:	0.907	0.898		0.966	0.971	0.902	0.971	0.971	0.961	0.971
**4**	AAR02639_high_6N5A.pdb:	0.917	0.912	0.966		0.995	0.917	0.985	0.985	0.985	0.985
**5**	ABI94581_high_6N5A.pdb:	0.917	0.917	0.971	0.995		0.922	0.980	0.980	0.980	0.980
**6**	AGB51388_high_6N5A.pdb:	0.883	0.976	0.902	0.917	0.922		0.902	0.902	0.902	0.902
**7**	AGU69978_high_6N5A.pdb:	0.902	0.898	0.971	0.985	0.980	0.902		1.000	0.980	0.990
**8**	AHF20373_high_6N5A.pdb:	0.902	0.898	0.971	0.985	0.980	0.902	1.000		0.980	0.990
**9**	EPI168665_high_6N5A.pdb:	0.912	0.898	0.961	0.985	0.980	0.902	0.980	0.980		0.980
**10**	EPI623939_high_6N5A.pdb:	0.912	0.898	0.971	0.985	0.980	0.902	0.990	0.990	0.980	
				**RMSD**							
	**structure:**	**1**	**2**	**3**	**4**	**5**	**6**	**7**	**8**	**9**	**10**
**1**	AAA43152_high_6N5A.pdb:		0.147	0.170	0.160	0.128	0.138	0.204	0.204	0.158	0.141
**2**	AAA56803_high_6N5A.pdb:	0.147		0.107	0.127	0.115	0.127	0.140	0.140	0.145	0.119
**3**	AAC54377_high_6N5A.pdb:	0.170	0.107		0.114	0.124	0.135	0.106	0.106	0.130	0.103
**4**	AAR02639_high_6N5A.pdb:	0.160	0.127	0.114		0.127	0.125	0.124	0.124	0.131	0.117
**5**	ABI94581_high_6N5A.pdb:	0.128	0.115	0.124	0.127		0.108	0.133	0.133	0.107	0.085
**6**	AGB51388_high_6N5A.pdb:	0.138	0.127	0.135	0.125	0.108		0.161	0.161	0.130	0.107
**7**	AGU69978_high_6N5A.pdb:	0.204	0.140	0.106	0.124	0.133	0.161		0.000	0.132	0.116
**8**	AHF20373_high_6N5A.pdb:	0.204	0.140	0.106	0.124	0.133	0.161	0.000		0.132	0.116
**9**	EPI168665_high_6N5A.pdb:	0.158	0.145	0.130	0.131	0.107	0.130	0.132	0.132		0.090
**10**	EPI623939_high_6N5A.pdb:	0.141	0.119	0.103	0.117	0.085	0.107	0.116	0.116	0.090	
				**Q-score**							
	**structure:**	**1**	**2**	**3**	**4**	**5**	**6**	**7**	**8**	**9**	**10**
**1**	AAA43152_high_6N5A.pdb:		0.998	0.997	0.997	0.998	0.998	0.995	0.995	0.997	0.998
**2**	AAA56803_high_6N5A.pdb:	0.998		0.999	0.998	0.999	0.998	0.998	0.998	0.998	0.998
**3**	AAC54377_high_6N5A.pdb:	0.997	0.999		0.999	0.998	0.998	0.999	0.999	0.998	0.999
**4**	AAR02639_high_6N5A.pdb:	0.997	0.998	0.999		0.998	0.998	0.998	0.998	0.998	0.998
**5**	ABI94581_high_6N5A.pdb:	0.998	0.999	0.998	0.998		0.999	0.998	0.998	0.999	0.999
**6**	AGB51388_high_6N5A.pdb:	0.998	0.998	0.998	0.998	0.999		0.997	0.997	0.998	0.999
**7**	AGU69978_high_6N5A.pdb:	0.995	0.998	0.999	0.998	0.998	0.997		1.000	0.998	0.999
**8**	AHF20373_high_6N5A.pdb:	0.995	0.998	0.999	0.998	0.998	0.997	1.000		0.998	0.999
**9**	EPI168665_high_6N5A.pdb:	0.997	0.998	0.998	0.998	0.999	0.998	0.998	0.998		0.999
**10**	EPI623939_high_6N5A.pdb:	0.998	0.998	0.999	0.998	0.999	0.999	0.999	0.999	0.999	

## Data Availability

Not applicable.
